# SCHFI 6.2 Self-Care Confidence Scale - Brazilian version: psychometric analysis using the Rasch model*

**DOI:** 10.1590/1518-8345.3378.3313

**Published:** 2020-08-31

**Authors:** Diná de Almeida Lopes Monteiro da Cruz, Ana Maria Miranda Martins Wilson, Michele Nakahara Melo, Ana Paula da Conceição, Leidy Johanna Rueda Diaz

**Affiliations:** 1 Universidade de São Paulo, Escola de Enfermagem, São Paulo, SP, Brazil.; 2 Universidade Federal de Minas Gerais, Escola de Enfermagem, Belo Horizonte, MG, Brazil.; 3 Instituto Dante Pazzanese de Cardiologia, São Paulo, SP, Brazil.; 4 Universidad Industrial de Santander, Bucaramanga, Colombia.

**Keywords:** Validation Study, Surveys and Questionnaires, Self Care, Patient Education as Topic, Heart Failure, Nursing, Estudos de Validação, Inquéritos e Questionários, Autocuidado, Educação de Pacientes como Assunto, Insuficiência Cardíaca, Enfermagem, Estudios de Validación, Encuestas y Cuestionarios, Autocuidado, Educación del Paciente como Asunto, Insuficiencia Cardíaca, Enfermería

## Abstract

**Objective:**

to evaluate the psychometric properties of the Self-Care Confidence Scale in heart failure in the Brazilian version of the Self Care Heart Failure Index, version 6.2, using the Rasch model criteria.

**Method:**

secondary study, of psychometric analysis, using the Rasch model, of the six items of the scale. The sample consisted of 409 patients with heart failure undergoing outpatient treatment [mean age 57.9 (standard deviation = 11.6) years, 54.8% male].

**Results:**

of the total of six items, one *(“De maneira geral, você está confiante sobre estar livre dos sintomas de insuficiência cardíaca?”)* presented maladjustment to the model (Infit = 1.84 and Outfit = 1.99). After the exclusion of this item, the others showed a good fit, composed one dimension and explained 55% of the variance in the data; the categories of response to the items were adequate, the values of separation and reliability of person were 2.13 and 0.82, respectively, and Cronbach’s alpha was 0.87. Items of extreme difficulty were identified and there is no differential functioning of the items in relation to sex.

**Conclusion:**

with the exclusion of the first item, the Self-Care Confidence Scale showed good psychometric properties, with caution in interpreting the results of the six-item scale.

## Abstract (pt)

**Objetivo:**

avaliar as propriedades psicométricas da Escala de Confiança no Autocuidado na insuficiência cardíaca da versão brasileira do *Self-Care Heart Failure Index*, versão 6.2, usando os critérios do modelo Rasch.

**Método:**

estudo secundário, de análise psicométrica, pelo modelo de Rasch, dos seis itens da escala. A amostra foi constituída por 409 pacientes com insuficiência cardíaca em tratamento ambulatorial [idade média de 57,9 (desvio-padrão=11,6) anos, 54,8% do sexo masculino].

**Resultados:**

do total de seis itens, um (“De maneira geral, você está confiante sobre estar livre dos sintomas de insuficiência cardíaca?”) apresentou desajuste ao modelo (*Infit*=1,84 e *Outfit*=1,99). Após a exclusão desse item, os demais apresentaram bom ajuste, compuseram uma dimensão e explicaram 55% da variância nos dados; as categorias de resposta aos itens foram adequadas, os valores de separação e confiabilidade das pessoas foram 2,13 e 0,82, respectivamente, e o alfa de Cronbach foi 0,87. Foram identificados os itens de extremos de dificuldade e que não há funcionamento diferencial dos itens em relação a sexo.

**Conclusão:**

com a exclusão do primeiro item, a Escala de Confiança no Autocuidado apresentou boas propriedades psicométricas, recomendando-se cautela na interpretação dos resultados da escala com seis itens.

## Abstract (es)

**Objetivo:**

evaluar las propiedades psicométricas de la Escala de Confianza en el Autocuidado, de la insuficiencia cardíaca, de la versión brasileña del *Self Care Heart Failure Index* versión 6.2, usando los criterios del modelo Rasch.

**Método:**

estudio secundario de análisis psicométrica de los seis ítems de la escala del modelo de Rasch. La muestra fue constituida por 409 pacientes, con insuficiencia cardíaca, en tratamiento de ambulatorio [edad media de 57,9 (desviación estándar=11,6) años; 54,8% del sexo masculino].

**Resultados:**

del total de seis ítems, uno (“*De maneira geral, você está confiante sobre estar livre dos sintomas de insuficiência cardíaca?”)* presentó desajuste (*Infit*=1,84 y *Outfit*=1,99) en el modelo utilizado. Después de la exclusión de ese ítem, los demás presentaron buen ajuste, compusieron una dimensión y explicaron 55% de la varianza de los datos; las categorías de respuesta a los ítems fueron consideradas adecuadas; los valores de separación y confiabilidad de las personas fueron 2,13 y 0,82, respectivamente; y, el alfa de Cronbach fue 0,87. Fueron identificados los ítems extremos identificados como difíciles dificultad. No hubo funcionamiento diferencial de los ítems en relación al sexo.

**Conclusión:**

con la exclusión del primer ítem, la Escala de Confianza en el Autocuidado presentó buenas propiedades psicométricas; por esa razón, se recomienda tener cautela en la interpretación de los resultados de la escala con los seis ítems.

## Introduction

Heart failure (HF) is considered the cardiac syndrome with the highest rates of morbidity and mortality and the main cause of hospital admissions, with worldwide prevalence rates of 1% to 7%^(^ 1 ^-^ 2 ^)^. Adequate self-care in HF has positive effects on patients’ clinical outcomes^(^ 3 ^-^ 5 ^)^, and the confidence that the person has that he/she can do self-care is fundamental for proper self-care^(^ 6 ^-^ 9 ^)^.

The Self-Care Confidence Scale in HF is one of the Self-Care Heart Failure Index - Version 6.2 (SCHFI 6.2)^(^ 10 ^-^ 11 ^)^, which has been adapted for use in Brazil^(^ 12 ^)^ and, as its name suggests, it assesses the person’s confidence that he/she can handle the self-care to control HF. The Self-Care Confidence Scale starts with the statement *“De maneira geral, você está confiante sobre...”* and each of its six items presents a specific topic: “*1) estar livre dos sintomas de insuficiência cardíaca?”; “...2) seguir o tratamento recomendado; “...3) avaliar a importância de seus sintomas?”; “... 4) reconhecer alterações na saúde, caso elas ocorram?”; “...5) fazer algo que possa aliviar seus sintomas?”; “...6) avaliar se um medicamento funciona*?”^(^ 12 ^)^. Responses to each item vary in scores from one to four from “*não confiante”, “um pouco confiante”, “muito confiante”* to *“extremamente confiante”* in the Brazilian version^(^ 12 ^)^. The scores obtained from each of the six items are added together to produce a total score that reflects the person’s self-confidence to take care of themselves.

In the study of adaptation and validation of SCHFI 6.2 for Brazil, the authors used traditional analyses of psychometrics. However, more recent approaches, such as Rasch analyses, allow testing hypotheses about scales that cannot be tested by traditional psychometric analyses. For example, the Rasch model is useful for testing specific hypotheses about the dimensionality of items within a scale^(^ 13 ^)^.

In traditional psychometric analyses, it is assumed that all items in the set of items that make up a given scale measure the same dimension as the phenomenon in question. It is important to verify this assumption because, summing the items’ scores can only be valid when all items measure the same dimension^(^ 13 ^)^. That is, if, in the Self-Care Confidence Scale, one of the items does not measure the same dimension as the others, the interpretations made about the scores obtained with the scale are not valid.

The experience of the authors of this article with the use of SCHFI 6.2 has shown that patients, in general, have difficulties in responding to the Self-Care Confidence Scale because they often express doubts about how to respond to their items. This experience motivated the realization of this study, which aimed to produce new evidence about the psychometric properties of the referred scale.

Considering that the Rasch model facilitates the identification of weaknesses in measurement instruments that cannot be detected by traditional psychometric analyses^(^ 14 ^)^, the objective of this study was to evaluate the psychometric properties of the Self-Care Confidence Scale in HF of the Brazilian version of SCHFI 6.2, using the Rasch model criteria. The tested hypotheses were that: 1) all items on the scale reflect the same dimension; 2) all items fit the Rasch model; 3) the scale allows good discrimination of degrees of confidence in self-care; 4) scale items do not vary in measurement between sex.

## Method

This is a methodological study, of psychometric analysis,^(^ 15 ^)^ of data from 409 patients with HF undergoing outpatient follow-up who responded to the Brazilian version of SCHFI 6.2 in another study^(^ 16 ^)^. The primary study sample was convenient and the inclusion criteria were: confirmed medical diagnosis of HF, with functional class I, II or III and clinical conditions that allowed participating in interviews. The exclusion criteria were the presence of psychiatric, oncological, infectious diseases, with repercussions in the general state, or endocrine-metabolic diseases without treatment (for example, Diabetes Mellitus or thyroid disease); recent postoperative period (up to 60 postoperative days) of any surgical intervention^(^ 16 ^)^.

The primary study obtained a favorable opinion from the Research Ethics Committee (Protocol No. 145,630 - 2012) and all participants signed the Free and Informed Consent Term (FICT). Other methodological procedures are detailed in the article that reports the primary study^(^ 16 ^)^.

From the primary study, demographic data and responses to the SCHFI Self-Care Confidence Scale, version 6.2 adapted for use in Brazil^(^ 17 ^)^. There were no missing data in the responses to this scale.

Rasch analysis was applied using the Winstep 3.91.0 software and Andrich’s model was chosen, considering the assumption that all items have the same structure on the rating scale. Next, the order of the analyses:

1) The polarity of the items on the scale was examined to check for inverted items. If there were inverted items, it would be necessary to adjust their scores before other analyses;
2) An analysis of the functionality of the response categories was carried out, determining the fulfillment of the following statistical criteria for the optimization of the categories proposed by Linacre^(^ 18 ^-^ 20 ^)^:
  - A minimum of ten observations within each category of the scale. Low counts within a category can lead to inaccurate estimates or instability in step calibrations;
  - The average of the measure should increase as the response categories increase;
  - Outfit Mean Square values must be between 0.5 and two. Values greater than two reveal a high mismatch, as well as values below 0.5 indicate the possibility of approximate patterns to the deterministic response models;
3) The unidimensionality of the scale was evaluated by analyzing the main components of the model residuals. The principle of unidimensionality in the Rasch Model indicates that the variable refers to only one attribute^(^ 21 ^-^ 22 ^)^. The criterion was adopted to consider the scale as one-dimensional when the eigenvalue of the first contrast was ? 2.0. The matrix of correlations between item residuals was also evaluated to identify local dependency. The principle of local independence determines that the probability of success or failure in a given item must not be conditioned to success or failure in another item. Therefore, the items must be independent of each other and the probabilities of error or success must have no relation between the items^(^ 23 ^)^. Correlations above 0.30 may indicate local dependence and possible violations of one-dimensionality;
4) The fit of the items to the model was investigated using the average square (MnSq) of the Infit and Outfit of the items. For this study, the cutoff values used were 0.7 (minimum) and 1.3 (maximum), as suggested by Wright^(^ 24 ^-^ 26 ^)^. The fit in the Rasch model (fit statistics) indicates whether the data deviate or not from the model^(^ 23 ^)^; provides the comparison between what was expected in the model and what was obtained with the sample^(^ 18 ^,^ 23 ^)^. For this, the results of Infit and Outfit are analysed with the results presented in the form of MnSq. Very high MnSq value may indicate erratic scores on the item. MnSq value <0.7 indicates little variability of scores on the item or very predictable response pattern^(^ 11 ^,^ 14 ^)^;
5) The reliability of the scale was assessed using the person reliability, Cronbach’s alpha and the person and item separation index. The reliability of person is conceptually equivalent to Cronbach’s alpha and a value above 0.80 is considered appropriate. In the case of Cronbach’s alpha, a value above 0.70 is appropriate. The person separation index indicates how many groups with different skill levels (confidence in self-care) the item allows to identify^(^ 27 ^)^. The item separation index indicates how many levels of difficulty the items are distributed in. A clinically useful tool should divide participants into at least two skill levels (high and low), as well as items must be distributed across at least three levels of difficulty;
6) The presence of the differential item functioning (DIF) was investigated to test the measurement invariance between the sexes at the item level, that is, which items for men are easier or more difficult than for women to fulfill the item description^(^ 28 ^)^. The cutoff point commonly used to indicate substantial DIF is the DIF contrast >0.5^(^ 29 ^)^, with a statistical significance <0.05 in the Rasch-Welch test;
7) An item was removed that presented mismatch according to the criteria mentioned above. After removing this item, the scale’s unidimensionality, the adjustment of the items, the separation and the Rasch reliability of the calibrated scale were checked again;
8) Finally, the map of the distribution of the sample and items in the same continuum (persons-items map) with the original scale and the calibrated scale was examined. On this map, persons’ ability and item difficulty are placed in the same metric unit^(^ 30 ^)^ and allow the researcher to identify the magnitude of the skill the item measures and whether the items are evenly distributed. The empirical map resulting from the Rasch analysis can be used as evidence of the instrument’s construct validity ^(^ 27 ^)^.

The unit of measurement used in the Rasch model is the logit (log-odds), which is a linear function of the probability of obtaining a score by a person who has a certain skill^(^ 21 ^)^. Zero on the logit scale represents, in Rasch’s analysis, arbitrarily, the mean; the easiest items have negative values and the most difficult items have positive values on the scale^(^ 22 ^)^.

## Results

Data from 409 HF patients with a mean age of 57.9 years (standard deviation = 11.6) were analysed, 248 (60.6%) of whom were white, with a mean schooling of 6.1 years (standard deviation = 4.1); 264 (64.5%) had marital coexistence and 314 (76.8%) were inactive in relation to the labor situation. Regarding clinical data, non-ischemic cardiomyopathy as a cause of HF was responsible for 366 (88.8%) of the cases, the mean ventricular ejection fraction was 40.2% (standard deviation = 13.6%) and 191 (46.7%) were functional class II. The average time of experience with HF was 64.6 months (standard deviation = 65.1) and the average score on the Self-Care Confidence Scale in HF was 56.5 (standard deviation = 21.6).

The results of the Rasch analysis of the responses of 409 patients with HF to the six items of the Self-Care Confidence Scale in HF showed that all items had positive polarity, with item-total correlations between 0.56 and 0.80.

The analysis of the functionality of the response categories showed that they meet all the established criteria, as shown in Table 1.

**Table 1:** Functionality of the response categories of the Self Care Heart Failure Index 6.2 Self-Care Confidence Scale - Brazilian version (N = 409). São Paulo, SP, Brazil, 2019

Category		Observations		Averages		Infit MnSq*	Outfit MnSq*	Andrich Threshold	Category measures
Number	Score	n	%	Observed	Expected				
1	1	281	11	-1.37	-1.82	1.56	1.77	Nenhum	-3.43
2	2	633	26	-1.00	-0.70	0.71	0.70	-2.20	-1.40
3	3	1097	45	0.75	0.71	0.75	0.75	-0.56	1.14
4	4	443	18	2.47	2.35	0.99	0.93	2.76	3.89

* MnSq = Mean square.

Table 1 shows that the frequencies and the distribution of the categories are adequate, the average of the observations increases as the scores of the response categories increase, Infit and Oufit, for all categories, are less than two and close to one and the item’s difficulty values (Andrich thresholds) also increase as the scores increase, indicating that each category is the most likely for a specific range of the construct continuum.

The analysis of the unidimensionality by main components of the residuals showed that the Rasch dimension explained 50% of the variance in the data. The analysis of the first contrast indicated that the eigenvalue was 1.6, with a second dimension explaining more than 14% of the variance. All correlations between item residuals were less than 0.1.

Analyses of the measurement values showed that item 1 *(“De maneira geral, você está confiante sobre... estar livre dos sintomas de insuficiência cardíaca?”)* did not fit the model. It was deleted and further analysis was done with the remaining five items. Table 2 shows the measurement values obtained, in descending order, with the respective scale adjustment parameters with six items.

**Table 2:** Adjustment measures for the items in the Self Care Heart Failure Index 6.2 Self-Care Confidence Scale - Brazilian version with six items (N = 409). São Paulo, SP, Brazil, 2019

Item	Total score	Model			Infit			Outfit			Item-total correlation coefficient	
		Measure	Standard error		MnSq*	ZStd†		MnSq*	ZStd†		Observed	Expected
1	976	0.90	0.08		1.84	9.9		1.99	9.9		0.56	0.76
5	1016	0.62	0.08		1.00	0.1		0.99	-0.1		0.77	0.75
6	1159	-0.41	0.09		0.91	-1.3		0.87	-1.8		0.74	0.72
3	1104	0.00	0.08		0.81	-2.9		0.81	-2.7		0.78	0.73
2	1234	-0.99	0.09		0.72	-4.2		0.75	-3.6		0.75	0.69
4	1121	-0.12	0.09		0.68	-5.0		0.66	-5.1		0.80	0.73
Average	1101.7	0.00	0.09		0.99	-0.6		1.01	-0.6			
Standard deviation	85.9	0.63	0.00		0.40	5.0		0.45	4.9			

* MnSq = Mean Square;

† ZStd = Standardized fit statistics

Table 2 shows that item 1 *(“De maneira geral, você está confiante sobre... estar livre dos sintomas de insuficiência cardíaca?*”) presented an important mismatch to the model. The infit and outfit values of item 1 were 1.84 and 1.99, respectively, remaining outside the cutoff values adopted, as described in the method (acceptable values of infit and outfit between 0.7 and 1.3). Table 3 presents the results of the scale with five items after removing item 1.

**Table 3:** Adjustment measures for the items of the Self Care Heart Failure Index 6.2 Self-Care Confidence Scale - Brazilian version with five items (N = 409). São Paulo, SP, Brazil, 2019

Item	Total score	Model			Infit			Outfit			Item-total correlation coefficient	
		Measure	Standard error		MnSq*	ZStd†		MnSq*	ZStd†		Observed	Expected
5	1016	1.07	0.10		1.24	3.1		1.26	3.2		0.79	0.83
3	1104	0.24	0.10		0.90	-1.4		0.88	-1.5		0.83	0.81
4	1121	0.07	0.10		0.78	-3.1		0.72	-3.7		0.83	0.80
6	1159	-0.30	0.10		1.07	0.9		1.01	0.2		0.78	0.79
2	1234	-1.08	0.10		0.94	-0.8		1.00	0.0		0.77	0.76
Average	1101.7	0.00	0.10		0.98	-0.3		0.97	-0.4			
Standard deviation	85.9	0.70	0.00		0.16	2.1		0.18	2.2			

* MnSq - Mean Square;

† ZStd - Standardized fit statistics

It can be seen in Table 3 that, without item 1, in the scale with five items, there are no items with Infit/Outfit values greater than 1.3 or less than 0.7, showing that all fit well to the model.

The result of the Rasch analysis for the scale with six items showed a separation of person and items of 1.93 and 6.78, respectively, and a reliability of person of 0.79 and item of 0.98, being that Cronbach’s alpha was 0.84. The values of separation and reliability of person on the scale with five items improved in relation to the analysis of the scale with six items, with the separation of person equal to 2.13, the reliability of person of 0.82 and the Cronbach alpha of 0.87. Nevertheless, the separation and reliability of items were the same obtained in the analysis of the scale with six items (6.78 and 0.98, respectively).

Table 4 shows the results of the analysis of the differential functioning of the items in relation to sex.

**Table 4:** Differential functioning of items in relation to sex of the Self Care Heart Failure Index 6.2 Self-Care Confidence Scale - Brazilian version (N = 409). São Paulo, SP, Brazil, 2019

Item	DIF* Contrast	Rasch-Welch	
		t†	Probability
1	-0.08	-0.49	0.62
2	0.09	0.51	0.61
3	0.00	0.00	1.00
4	0.23	1.35	0.17
5	-0.14	-0.83	0.40
6	-0.07	-0.40	0.69

* DIF = Differential Item Functioning);

† t- test t

It can be seen, in Table 4, that none of the six items showed substantial contrast (all were less than 0.5) between men and women using the Rasch-Welch test.

As for the unidimensionality, the analysis by main components of the residuals showed that, for the scale with five items, the Rasch dimension explained 55% of the variance in the data. The analysis of the first contrast indicated that the eigenvalue was 1.4, with a second dimension explaining more than 13% of the variance. The correlations between the residuals of the items showed an absence of local dependence, as they were all less than -0.10.

**Figure 1:**
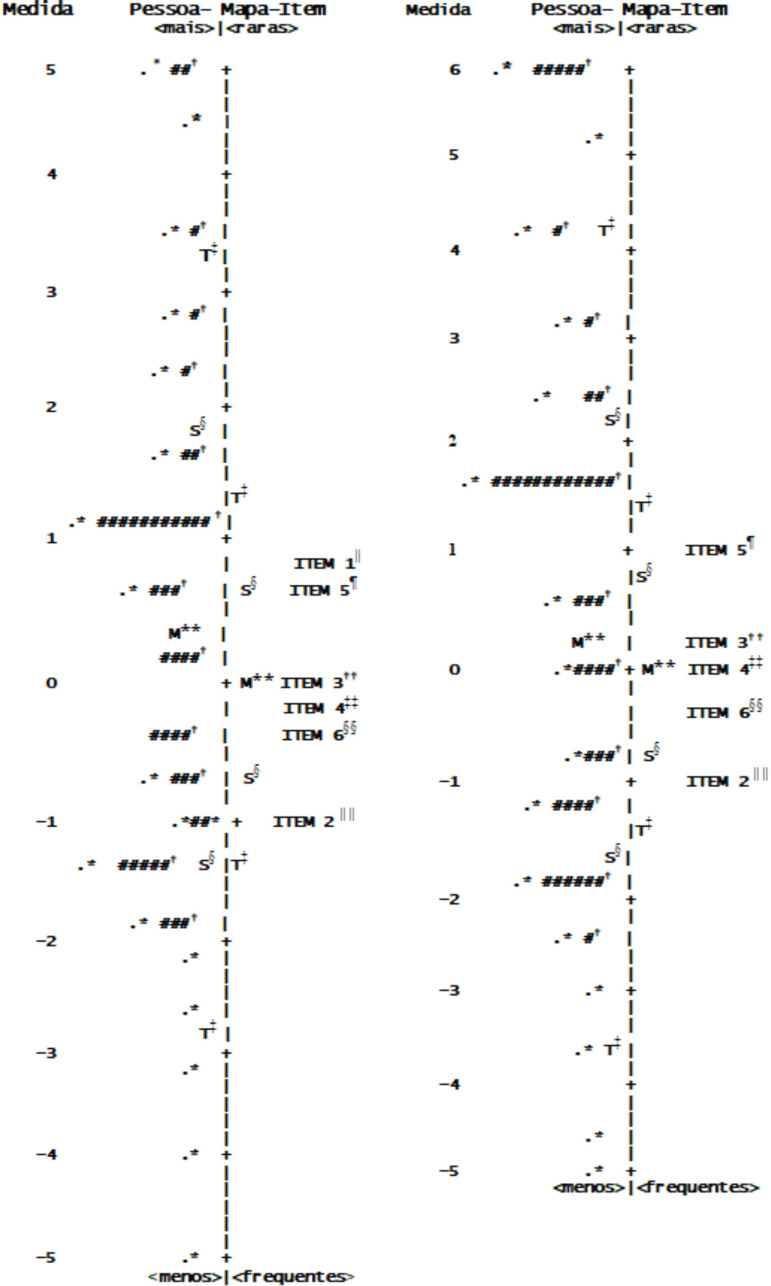
Person-item map for the Self-Care Confidence Scale with six items (left) and five items (right) (N = 409). São Paulo, SP, Brazil, 2019 * = one or seven persons; †# = “each #”: eight persons; ^‡^T = two standard deviations; ^§^S = one standard deviation; ^||^ ITEM 1 =... be free from the symptoms of heart failure; ^¶^ITEM 5 = ... do something that can relieve your symptoms?; **M = Average; ^††^ITEM 3 =... assess the importance of your symptoms?; ^‡‡^ITEM 4 =... recognize changes in health if they occur?; ^§§^ITEM 6 =... assess whether a drug works?; ^|||||^ITEM 2 =... follow the recommended treatment?

Figure 1 shows two maps of person-items. One (left) is the person-item map for the six-item scale and the other (right) is the map for the five-item scale. Each of the person-item maps shows the distribution of the difficulty of the items on the right and the distribution of the skills of the person on the left. The top represents the most difficult items and the most skillful participants (more confidence in self-care). On the other hand, the bottom represents the easiest items and the participants with the least ability (less confidence in self-care). Both person (indicated by the # sign) and items are distributed on the same vertical continuum where the measurements are in logits and the zero point is the midpoint.

The item map (Figure 1) was examined to identify whether the item hierarchy was consistent with the theory. For the scale with six and with five items, it is observed that most items were approximately between -1 logit and +1 logit. Regarding person, the distribution is more dispersed and covered the approximate range of -5 logit to +6 logit.

For the six-item scale, the question “*De maneira geral, você está confiante sobre... estar livre dos sintomas de insuficiência cardíaca?”* was the most difficult (Figure 1). In the case of the five-item scale, the question “*De maneira geral, você está confiante sobre... fazer algo que possa aliviar seus sintomas*?” was the most difficult (Figure 1). In both cases, the item “*De maneira geral, você está confiante sobre... seguir o tratamento recomendado?”* was the easiest (Figure 1).

## Discussion

The analyses carried out made it possible to investigate the structural validity and reliability of the Self-Care Confidence Scale of the Brazilian version of SCHFI 6.2^(^ 12 ^)^, as well as identifying the degree of difficulty of each item and the adequacy of the categories of responses to the items.

The results obtained indicate that the instrument has a good functioning if item 1 is excluded (“*De maneira geral, você está confiante sobre... estar livre dos sintomas de insuficiência cardíaca?*”). This item presented a maladjustment to the model, because the values of Infit MnSq (1.84) and Outfit MnSq (1.99) (Table 2) are outside the acceptable limits (from 0.7 to 1.3). When the analysis indicates that an item does not fit the model, it means that the item is not related to the construct under study^(^ 18 ^)^. In the case of this study, it can be said that item 1 is not related to the construct of Confidence in Self-Care as are the other items^(^ 31 ^)^.

This result is possibly due to the Portuguese version of this item. Although adaptation procedures have been rigorous^(^ 12 ^,^ 17 ^)^, the analyses that were carried out did not allow the verification of the functioning of each item in the set of the scale. It is suspected that the source of the mismatch of the item is the Portuguese language version as follows: in the original version of the instrument, the item is “In general, how confident are you that you can…. keep yourself free of heart failure symptoms?”; in the Brazilian version, the item was *“De maneira geral, você está confiante sobre... estar livre dos sintomas de insuficiência cardíaca?”.* In the experience of the authors of the study reported here, patients have difficulty in answering this item and often understand it as something related to faith or the hope that they will be well in the future, without linking “being free from the symptoms of heart failure” to something they can do themselves. These informal observations suggest that the Brazilian version of item 1 lacked the idea of “... that you can ...” - *que você consegue* - which is explicit in the original version.

To improve the Brazilian version of the item, it would be necessary to include the idea of “*conseguir*”, as, for example, *“De maneira geral, quão confiante você está de que você consegue...”*. With the item as it is (“*De maneira geral, você está confiante sobre... estar livre dos sintomas de insuficiência cardíaca*?”), it may be more difficult for the respondent to learn that what you want to know is trust in what he can do and not trust in a future state.

In the study of adaptation of the instrument^(^ 17 ^)^, the author mentions that the statement of the Self-Care Confidence Scale changed after the evaluation by the committee of judges. The term “confident”, from the original, would have been translated as “*seguro*” and then changed to “*confiante*”, with no other item being changed by the committee of judges or due to the pre-test carried out with 30 patients^(^ 17 ^)^. The adapted SCHFI 6.2 was applied to 190 patients with HF and the data were submitted to internal consistency analysis. Cronbach’s alpha of the Self-Care Confidence Scale was 0.94, which is very good, but the results showed that if item 1 were excluded, this index would not change^(^ 17 ^)^, indicating that this item was no longer contributing to the internal consistency of the scale. Rasch’s analysis, which offers more information on the suitability of items, revealed that this item needs to be improved if kept.

As for the other properties of the scale, the results show that the Brazilian version of the SCHFI 6.2 Self-Care Confidence Scale with six or five items is one-dimensional, explaining 50% or 55% of the variance in the data, respectively, with the eigenvalue of the first contrast less than two. These results appear to be consistent with what was obtained in the validation study of the Brazilian version of SCHFI 6.2^(^ 12 ^)^ in which confirmatory factor analysis, used to test a model with three components, showed the Self-Care Confidence Scale as one of the three components of SCHFI 6.2. However, there are different results in other studies that also used confirmatory factor analysis^(^ 11 ^,^ 32 ^)^. In a study that explored the dimensionality and reliability of SCHFI 6.2, in its original English version, with data from 629 adults with HF, it was demonstrated that the Self-Care Confidence Scale is one-dimensional, confirming what had been assumed in other studies^(^ 32 ^)^. However, confirmatory factor analysis in another study, conducted with data from 659 Italian HF patients, revealed the Self-Care Confidence Scale with two factors, called Basic Self-Care Confidence and Advanced Self-Care Confidence. The factor of Basic Confidence in Self-Care included the most general items (for example: “...*seguir o tratamento recomendado*”?), while Advanced Confidence in Self-Care reflected more challenging behaviors (for example: “... *estar livre dos sintomas de insuficiência cardíaca?”),* which require specific guidance and experience^(^ 11 ^)^. The discrepancy between the two studies that used confirmatory factor analysis was attributed to the differences in method with which the analyses were performed and not to differences between the samples^(^ 11 ^)^. The results of the study in which the Self-Care Confidence Scale resulted with two factors were supported by more robust techniques (cross-validation) and resulted in models with better adjustment rates^(^ 11 ^)^. In addition, the final analyses were made considering the three scales separately^(^ 11 ^)^ and not as a single model as in the other studies cited^(^ 12 ^,^ 32 ^)^. The results of this study showed that the scale is one-dimensional. However, as far as it is known, it is the only study that used Rasch analysis to investigate the properties of the Self-Care Confidence Scale. These controversial results indicate that the dimensionality of the Self-Care Confidence Scale needs further study.

Another property that Rasch’s analysis allowed to verify is whether the response scale of each item is adequate. In the case of the Brazilian version of the Self-Care Confidence Scale, the responses to each item vary in scores from one to four (1 *=“não confiante”; 2=“um pouco confiante”; 3=“muito confiante” e 4=“extremamente confiante*”)^(^ 12 ^)^. The data in Table 1 show that the four categories of responses (*não confiante; um pouco confiante; muito confiante; extremamente confiante*) represent increasing intensities of confidence, since the average of category 1 is lower than that of category 2, which in turn , is less than category 3, and less than category 4.

Other important information provided by Rasch’s analysis was the DIF (Table 4) and the degree of difficulty of the items (Figure 1). The DIF evaluation allowed establishing that the instrument does not present bias due to the sex of the respondents.

Regarding the degree of difficulty of the items, it can be seen, in figure 1, that it is the map of person-items of the scale with six and five items, that item 1 (“*De maneira geral, você está confiante sobre... estar livre dos sintomas de insuficiência cardíaca?”)* it was the most difficult item. With the exclusion of item 1, item 5 (“*De maneira geral, você está confiante sobre... fazer algo que possa aliviar seus sintomas?*”) becomes the most difficult, without changing the order of difficulty of the others items (Figure 1). In descending order of difficulty, after item 5, come the items: 3 *(“De maneira geral, você está confiante sobre... ...avaliar a importância de seus sintomas?”);* 4 (“*De maneira geral, você está confiante sobre... ... reconhecer alterações na saúde, caso elas ocorram?”); 6 (“De maneira geral, você está confiante sobre... ...avaliar a importância de seus sintomas?”)* And 2 (*“De maneira geral, você está confiante sobre... ...seguir o tratamento recomendado?”*), which was the easiest item. This order of difficulty seems reasonable, considering the challenges that the represented behaviors offered to patients with HF. Having confidence that they can do something to alleviate the symptoms seems to be more difficult than having confidence that you can assess the importance of the symptoms. Having confidence that they can assess the importance of symptoms seems more challenging than having confidence that they can recognize them. Given these behaviors, it seems that having confidence that you can follow the recommended treatment is the least challenging behavior for patients with HF. Knowing the degree of difficulty of the items can serve as a guide for the organization of self-care promotion programs for people with HF.

The results regarding the distribution of persons (Figure 1) suggest that there is a need for items to measure difficulties greater than those of item five and less than those of item 2, because there are parts of the distribution of persons that are outside the spectrum of difficulties of existing items. It is also observed that new items would fit with difficulties between items 2 and 6 and between items 3 and 5.

Further studies on the properties of the SCHFI 6.2 Self-Care Confidence Scale, in its various versions, using Rasch analysis, are necessary to refine an instrument that assesses a fundamental variable for knowledge about self-care in HF. In the case of the Brazilian version, the first step would be to review the version of item 1 through content validity studies^(^ 14 ^)^. If, in a new version, this item fits the model, comparisons between samples of Brazilians and samples that used the original instrument will be favored.

The main limitation of the reported study is the fact that the data are from patients from a single public service specialized in Cardiology, which indicates caution in generalizing the results. Studies that analyse whether there is a differential functioning of items between patients with HF from specialized and non-specialized services would offer evidence on the magnitude of such limitations.

## Conclusion

In summary, the analysis using the Rasch method of the Brazilian version of the SCHFI 6.2 Self-Care Confidence Scale allows us to conclude that: 1) all items on the scale reflect the same dimension; the scale is one-dimensional; 2) only item 1 *(“De maneira geral, você está confiante sobre... estar livre dos sintomas de insuficiência cardíaca?”)* it did not fit the Rasch model, and caution was recommended when interpreting the scale scores with the six items; 3) the scale allows good discrimination of degrees of confidence in self-care; 4) scale items do not vary in measurement between sexes.
